# Generation of a Tenascin-C-CreER2 Knockin Mouse Line for Conditional DNA Recombination in Renal Medullary Interstitial Cells

**DOI:** 10.1371/journal.pone.0079839

**Published:** 2013-11-11

**Authors:** Wenjuan He, Qionghong Xie, Yingying Wang, Jing Chen, Min Zhao, Linda S. Davis, Matthew D. Breyer, Guoqiang Gu, Chuan-Ming Hao

**Affiliations:** 1 Division of Nephrology, Huashan Hospital, Fudan University, Shanghai, China; 2 Gladstone Institute of Virology & Immunology, San Francisco, California, United States of America; 3 Nephrology Division, Vanderbilt University Medical Center School of Medicine, Nashville, Tennessee, United States of America; 4 Biotechnology Discovery Research, Lilly Research Laboratories, Eli Lilly and Company, Indianapolis, Indiana, United States of America; 5 Department of Cell and Developmental Biology, Vanderbilt University Medical Center, Nashville, Tennessee, United States of America; Emory University, United States of America

## Abstract

Renal medullary interstitial cells (RMIC) are specialized fibroblast-like cells that exert important functions in maintaining body fluid homeostasis and systemic blood pressure. Here, we generated a RMIC specific tenascin-C promoter driven inducible CreER2 knockin mouse line with an EGFP reporter. Similar as endogenous tenascin-C expression, the reporter EGFP expression in the tenascin-C-CreER2^+/−^ mice was observed in the inner medulla of the kidney, and co-localized with COX2 but not with AQP2 or AQP1, suggesting selective expression in RMICs. After recombination (tenascin-C-CreER2^+/−^/ROSA26-lacZ^+/−^ mice + tamoxifen), β-gal activity was restricted to the cells in the inner medulla of the kidney, and didn't co-localize with AQP2, consistent with selective Cre recombinase activity in RMICs. Cre activity was not obvious in other major organs or without tamoxifen treatment. This inducible RMIC specific Cre mouse line should therefore provide a novel tool to manipulate genes of interest in RMICs.

## Introduction

The renal medullary interstitial cells (RMIC) are a population of specialized stroma-like cells in renal medulla. These cells, characterized by abundant cytoplasmic lipid droplets, are arranged in rows with their long axis perpendicular to adjacent tubules and vessels [1], [2]. In addition to supporting the renal structure, RMICs have been demonstrated to play important roles in the maintenance of body fluid homeostasis and normal systemic blood pressure. Animal studies show that chemical ablation of RMICs with BEA leads to systemic hypertension [3]. RMIC cyclooxygenase-2 (COX2) expression is also suggested to play important roles in renal response to stress, such as sodium loading and water deprivation [4]–[7]. To better understand the molecular basis of the physiological roles of RMICs, a Cre-recombinase/LoxP-based RMIC-specific gene deletion could be a powerful approach to investigate the significance of specific genes in RMICs. Here, we report an inducible RMIC-specific Cre-recombinase line under the control of endogenous tenascin-C promoter.

## Materials and Methods

### Animals

Ethics statement: Mice used in the present study were maintained in the animal facility of Vanderbilt University Medical Center, where they were housed in a constant temperature room with a 12-hour dark/12-hour light circle, and allowed free access to standard rodent chow and water. All animal studies were approved by the Institutional Animal Care and Use Committees of Vanderbilt University. C57Bl/6J wild type mice were obtained from Jackson Laboratories. ROSA26-lacZ reporter mice and genotyping methods were previously reported [8].

### Construction of the targeting vector

The homogenous recombination arms were derived from a BAC library (RPCI-22 mouse BAC library, Invitrogen). The targeting vector was assembled in pBlue and contains a 4 kb 5′ arm, an inducible CreER2, an IRES-EGFP (Clontech), a FRT flanked PGK-neo selection cassette and a 2 kb 3′ arm. The CreER2 was made from CreER version 1 (kindly provided by Dr. Andrew P. McMahon) via site directed mutagenesis according to literature [9].

### Screen ES cells by Southern Blot

Run digested DNA in 1% agarose gel. Take picture of agarose gel to be blotted with phosphorescent ruler lined up along side it, such that the ruler is lined up with the top of the wells. Depurinate the DNA in the gel by rocking it in 0.25 M HCl for exactly 10 min, and alkaline denature the gel in 0.4 M NaOH 3×15 min. While shake the gel in 20XSSC for 5 min, set up the blot from bottom to top: 1) A large dish filled with 20×SSC with glass plate on top of it to rest the gel. 2) Two pieces of wick- blotting paper cut to the width of the gel and length such that the wick is in contact with the bottom of the dish. Wet the wick with 20×SSC and smooth out the bubbles gently with a glass pipette. 3) Agarose gel, turned upside down, with a nick in the bottom right hand corner for orientation. Smooth out bubbles. Place plastic wrap to cover the entire gel and cut out the wrap around the gel such that the blot will not short-circuit. 4) Hybond N+ nylon membrane cut to the exact size of the gel, with a nick in the corner for orientation. Wet membrane with dH_2_O, place on top of gel and smooth it out. 5) Four pieces of blotting paper cut to size of the gel. Wet the first blotting paper with 20×SSC, put on top of blotting paper, and smooth out. Put other three on top. 6) Glass plate and additional weight to keep blot in place. Transfer overnight. The next day, take apart blot being careful not to remove the membrane from the gel. Take off the gel and membrane together, and flip. Use a pencil to mark the wells. Auto X-link membrane with Stratalinker. Prehybridize the membrane at 65°C for at least 1 h, add probes and hybridize at 65°C overnight. Wash the membrane 3×10 min at 65°C and then expose at −80°C for 4–7 days. Primers used for synthesizing 5′ probe were: 5′-TAGAGCAGGTGGTCCCAAACAT-3′ and 5′-CCAGGAGCCAGGAAATAGCCTTA-3′. Primers used for synthesizing 3′ probe were: 5′-GATGACGACTACACTGGGGAA-3′ and 5′-ACTGGGGCACCTTTGCTCTT-3′.

### PCR Genotyping

Mice were genotyped using genomic DNA isolated from tail biopsy. PCR primers used to amplify across the region where the 5′ CreER2 was inserted were: sense: 5′-GGGGGCAAGAAGGCAAAAAT-3′; antisense-1: 5′-GTTCTGCGGGAAACCATTT-3′; antisense-2: 5′-TCTCGCTTGTGCCTGATGAT-3′. Primer pair of sense and antisense-1 gave a band of ∼430 bp for a targeted allele and no band for a wild type allele. Primer pair of sense and antisense-2 gave a band of ∼300 bp for a wild type allele and no band for a targeted allele.

### In Situ Hybridization

In situ hybridization was performed as previously described [5]. Briefly, prior to hybridization, tissue sections were deparaffinized, refixed in 4% paraformaldehyde, treated with proteinase K (20 µg/ml), washed with PBS, refixed in 4% paraformaldehyde, and treated with triethanolamine plus acetic anhydride (0.25% vol/vol). Finally, sections were dehydrated with 100% ethanol. ^35^S-labeled antisense and sense riboprobes from mouse tenascin-C were hybridized to the sections at 55°C for 18 h. After hybridization, the sections were washed at 65°C once in 5X SSC (1X SSC is 0.15 M NaCl and 0.015 M sodium citrate, pH 7.0) plus 10 mM β-mercaptoethanol (BME), then once in 50% formamide, 2X SSC, and 100 mM BME for 30 min. After an additional two washes in 10 mM Tris, 5 mM EDTA, 500 mM NaCl (TEN) at 37°C, the sections were treated with RNase A (10 µg/ml) at 37°C for 30 min, followed by another wash in TEN at 37°C. Sections were then washed twice in 2X SSC and twice in 0.1X SSC at 65°C. Slides were dehydrated with graded ethanol containing 300 mM ammonium acetate. Photomicrographs were taken from slides dipped in K5 emulsion (Ilford Ltd., Knutsford, Cheshire, United Kingdom) diluted 1∶1 with 2% glycerol/water and exposed for 7 days at 4°C. After development in Kodak D-19, slides were counterstained with hematoxylin. Photomicrographs were taken with a Zeiss Axioskop microscope.

### Immunofluorescent Staining

The kidney tissues were first fixed in 4% paraformaldehyde and then incubated in 30% sucrose overnight. Cryostat sections (5 µm) were blocked with 3% normal donkey serum for 20 min, and then incubated with primary antibody for 60 min at room temperature. After washing in PBS, the sections were incubated in Cy2 or Cy3 conjugated anti-IgG secondary antibody (Jackson ImmunoResearch Laboratories, 1∶200) for 30 min. Sections were washed again in PBS for 5 times and microscopy was performed with a Zeiss Axioskop and spot-cam digital camera (Diagnostic Instruments) or confocal microscope (Zeiss LSM510). The primary antibodies used for immunofluorescence studies were: anti-aquaporin-1 (AQP1) antibody (Santa Cruz mouse monoclonal, 1∶100), anti-aquaporin-2 (AQP2) antibody (Santa Cruz goat polyclonal, 1∶400), anti-COX2 antibody (Cayman rabbit polyclonal, 1∶500).

### X-gal Staining

Frozen sections were freshly cut and fixed with cold formalin for 10 minutes at 4°C. After 3 changes of PBS wash for 5 minutes each, the slides were rinsed in distilled water and washed in β-gal wash buffer (0.1 M Phosphate buffer, 2 mM MgCl_2_, 5 mM EGTA, 0.01% Sodium Deoxycholate, 0.02% NP40, pH 7.3) for 10 minutes at RT. Then the slides were moved to X-gal stain solution (5 mM Potassium Ferrocyanide, 5 mM Potassium Ferricyanid, 1 mg/ml X-gal in β-gal wash solution) in a humidified chamber for 24 hours at 37°C. After 2 changes of PBS wash for 5 minutes each, the slides were rinsed with distilled water and mounted with aqueous mounting medium.

## Results and Discussion

**Figure 1:**
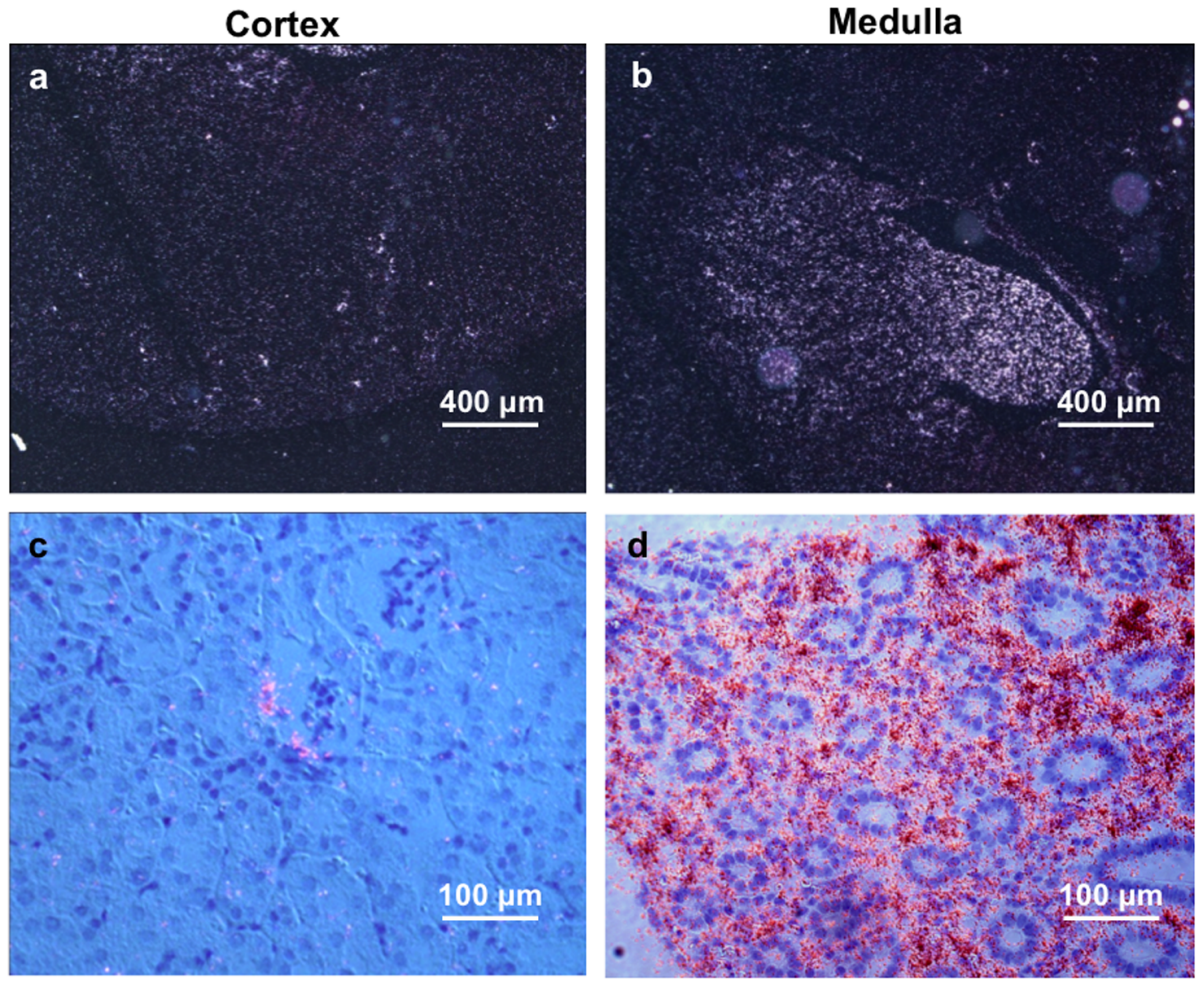
High levels of tenascin-C mRNA expression in the mouse renal medullary interstitium. (a, b) Dark field in situ hybridization pictures show high levels of tenascin-C mRNA in the mouse renal medulla with low levels in the renal cortex. Scale bar, 400 µm. (**c**, **d**) Bright field in situ hybridization pictures further show tenascin-C mRNA is highly expressed in the renal medullary interstitium. Scale bar, 100 µm.

Our microarray study identified tenascin-C as one of RMIC specific gene products in the kidney (Table 1). Selective expression of tenascin-C mRNA in the renal medullary interstitial cells was further confirmed by in situ hybridization (Figure 1). The data are consistent with published studies showing tenascin-C expression in the renal medullary stroma of the adult mice [10]–[12].

**Table 1:** Microarray reveals several genes that are selectively expressed in the renal medullary interstitial cells (RMICs).

Genes	Ratio
Mus musculus DAN	0.033
Mus musculus HIC-5 mRNA	0.042
Mus musculus GDP-dissociation in	0.049
Reticulocalbin	0.056
Tenascin-C	0.06
Retinol binding protein 1, cecul	0.087
Mus musculus lysyl oxidase-2	0.099
G-protein coupled receptor 26	0.101

Ratio represents the mRNA levels of a gene in inner medullary collecting ducts (IMCDs) as compared to that in renal medullary interstitial cells (RMICs).

Tenascin-C is one of the four members of the tenascin family (tenascin-C, -X, -R, -W) that encode glycoproteins found in the extracellular matrix [13]–[15]. Tenascin-C is abundantly expressed in the mesenchyme surrounding developing epithelia in virtually every organ during development. In contrast, its expression in normal adult tissues is very low or undetectable and highly restricted in stromal cells.

**Figure 2:**
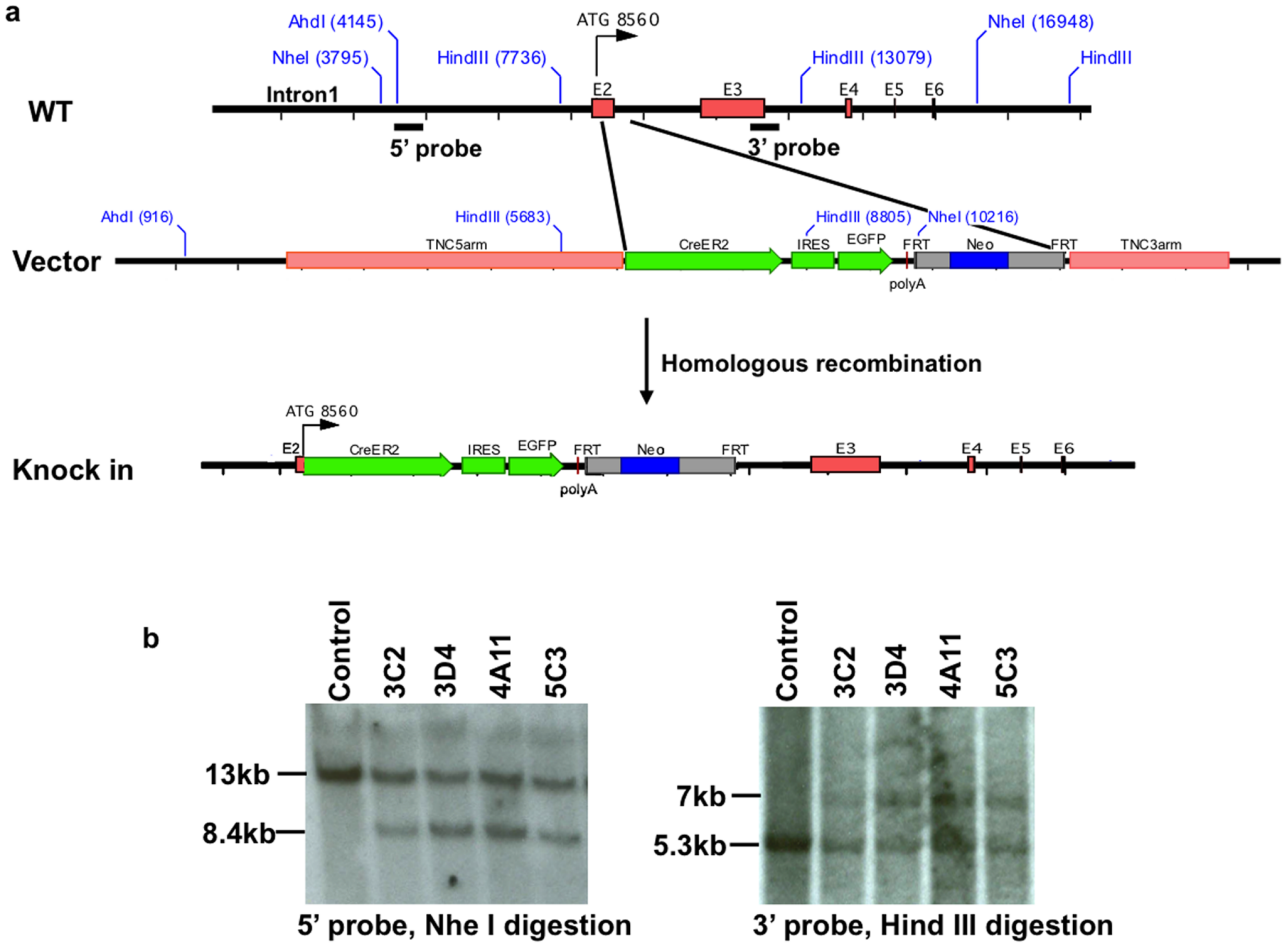
Generating Tenascin-C-CreER2 Mice. (a) Targeting strategy. The targeting construct has a 4 kb 5′ arm that locates immediately upstream of the tenascin-C gene ATG, an inducible CreER2, IRES-EGFP reporter, a PGK neo selection cassette flanked by FRT and a 2 kb 3′ arm that locates after exon2. (b) Southern blots show four targeted ES cell clones that give expected hybridization patterns (See Materials and Methods section for details). 5′ probe and 3′ probe are indicated in (a).

Considering that the mouse tenascin-C gene has a 31 kb intron1 (Figure 2a) that may contain important transcriptional regulatory elements, and heterozygous tenascin-C knockout mice have normal phenotypes [16], [17], a “knock-in” strategy was used to create a Cre-recombinase mouse driven by endogenous tenascin-C promoter. The Cre transgene was engineered immediately behind the translation start site (ATG) of the tenascin-C gene to ensure that the expression pattern of Cre is similar to that of the endogenous tenascin-C gene. Mouse genomic tenascin-C gene was obtained by a BAC library screen. The targeting construct included a 4 kb 5′ arm homogenous to 5′ region upstream of the tenascin-C gene translation start codon in exon 2, an inducible CreER2, an IRES (internal ribosome entrance site)-EGFP (enhanced green fluorescence protein), a PGK-Neo selection cassette flanked by FRT, and a 2 kb 3′ homogenous arm. The IRES-EGFP cDNA in the targeting construct allows for detecting site of Cre expression in mice carrying the transgene. The targeting construct was linearized by Ahd I and introduced into E14 129SvJ ES cells with established protocols [18] at Vanderbilt Mouse/ESC Shared Resource. Neo-resistant ES cell clones were screened by southern blot, and four correctly targeted clones were identified (Figure 2b). Two targeted clones (3D4 and 5C3) were picked for blastocyst injection. Germline transmission of the transgene was obtained from both of these two lines. Genotyping was achieved through PCR of tail DNA (Figure 2c). Mice heterozygous for the tenascin-C-CreER2 allele were viable and of normal size without significant developmental or functional abnormalities.

**Figure 3:**
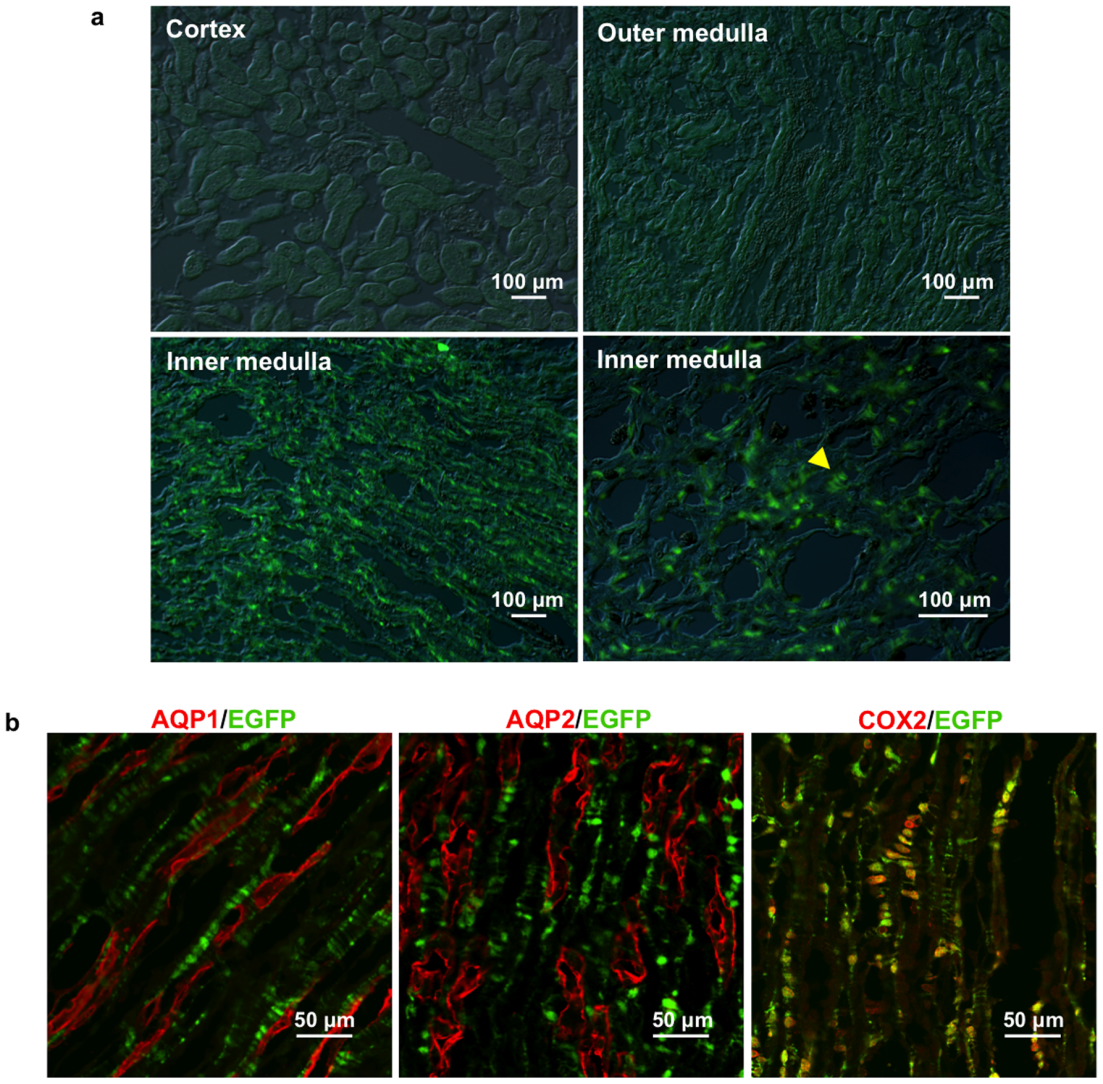
Exclusive EGFP expression in the renal medullary interstitial cells of tenascin-C-CreER2-EGFP mice. (a) EGFP expression was examined in the kidney of adult tenascin-C-CreER2-EGFP^+/−^ mice. Yellow arrow indicates EGFP positive cells arranged like rug of ladder resembling renal medullary interstitial cells. Scale bar, 100 µm. (b) Co-staining with renal structural markers (red). AQP1, thin limb of loop of henle; AQP2, collecting duct; COX2: renal medullary interstitial cell specific. Scale bar, 50 µm.

To test if the ectopic genes are expressed in the same pattern as endogenous tenascin-C gene, EGFP expression of the mice heterozygous for the tenascin-C-CreER2 allele was examined. Abundant EGFP expression was observed in the inner renal medulla but not in the cortex or outer medulla (Figure 3a). The EGFP-positive cells are arranged in rows, a typical morphology of renal medullary interstitial cells. Co-labeling studies showed that the EGFP did not co-localize with renal collecting duct marker AQP2 or descending thin limb marker AQP1, but co-localized with renal medullary interstitial cell specific COX2 (Figure 3b). This pattern is consistent with selective expression of EGFP in the renal medullary interstitial cells. No obvious EGFP was observed in other organs or tissues of adult mice examined including the heart, spleen, liver, skin and brain (data not shown).

**Figure 4:**
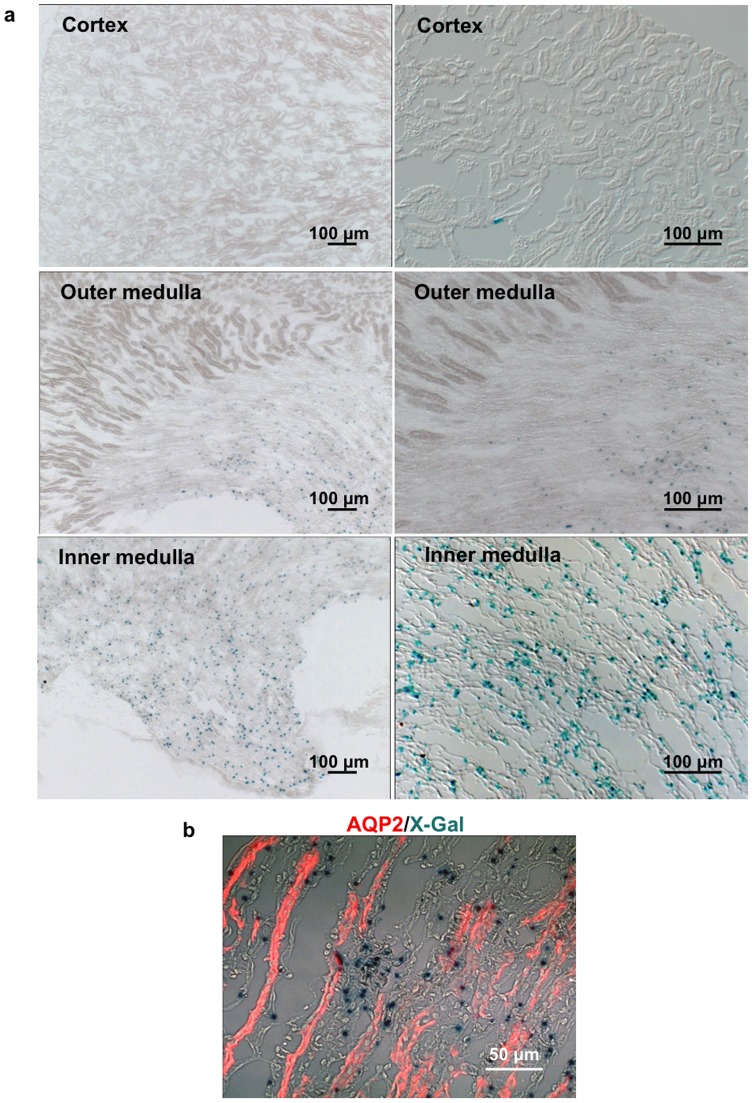
Exclusive β-gal activity in the renal medullary interstitial cells of tenascin-C-CreER2^+/−^/ROSA26-lacZ^+/−^ mice with tamoxifen injection. (a) X-gal staining shows β-gal activity in the kidney of tenascin-C-CreER2^+/−^/ROSA26-lacZ^+/−^ mice with tamoxifen injection. Scale bar, 100 µm. (b) X-gal stained renal medullary section co-stained with renal collecting duct marker AQP2 (red). Scale bar, 50 µm.

**Figure 5:**
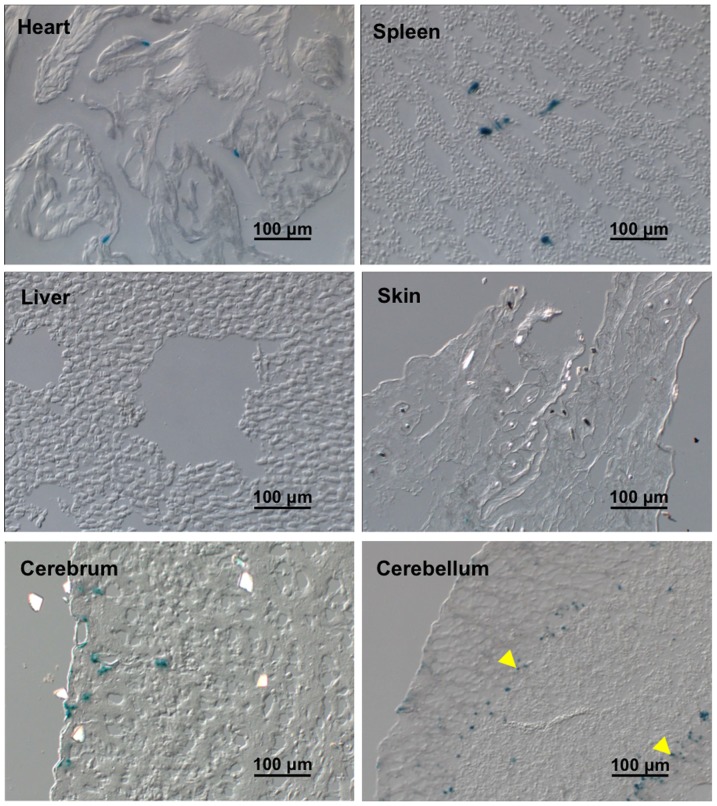
β-gal activity in major organs of tenascin-C-CreER2^+/−^/ROSA26-lacZ^+/−^ mice with tamoxifen injection. Pictures of X-gal staining shows no significant b-gal activity in the heart, spleen, liver, skin and cerebrum but in certain cells lining between different layers of cerebellum (indicated by yellow arrows) of tenascin-C-CreER2^+/−^/ROSA26-lacZ^+/−^ mice with tamoxifen injection. Scale bar, 100 µm.

**Figure 6:**
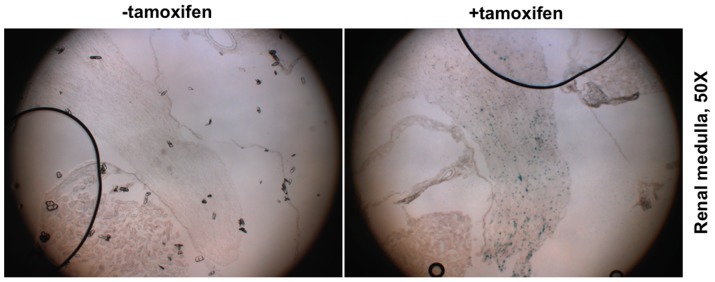
No leak of Cre recombinase activity in tenascin-C-CreER2^+/−^/ROSA26-lacZ^+/−^ mice without tamoxifen injection. X-gal staining show abundant β-gal activity in the renal medulla of tenascin-C-CreER2^+/−^/ROSA26-lacZ^+/−^ mice with tamoxifen injection but not in mice without tamoxifen injection.

ROSA26-lacZ reporter mice were used to monitor Cre activity by virtue of β-galactosidase (β-gal) expression that is dependent on Cre recombinasemediated release of a stop codon before β-gal cDNA. Tamoxifen (1.5 mg/25 g bw/day) was administered to tenascin-C-CreER^+/−^/ROSA26-lacZ^+/−^ mice at the age of 8 wks for continuous two weeks. Three days after the last injection, β-gal activity in major organs was examined by X-gal staining. The recombination reporter β-gal activity was highly restricted to the inner medulla of the kidney but not in the renal cortex (Figure 4a). Co-labeling with renal collecting duct marker AQP2 showed that the β-gal positive cells were localized in the inner medullary interstitium between collecting (Figure 4b). No significant β-gal activity was observed in the heart, spleen, liver, skin and cerebrum of the brain (Figure 5). However, there was detectable β-gal activity in some cells between different layers of cerebellum (Figure 5), consistent with previous studies showing tenascin-C expression in the forebrain and the cerebellum of the adult mice [19]. In addition, no β-gal activity was detected without tamoxifen treatment (Figure 6), suggesting no leak of the inducible Cre recombinase activity of the tenascin-C-CreER2^+/−^/ROSA26-lacZ^+/−^ mice.

In summary, we have generated a tenascin-C-CreER2-IRES-EGFP knockin mouse line, and demonstrated that this mouse line has EGFP expression and inducible Cre activity predominantly in the medullary interstitial cells of the kidney. The tenascin-C-CreER2 knockin mouse is therefore able to be utilized for introducing DNA recombination specifically in the renal medullary interstitial cells of the kidney. By inactivating genes of interest or driving ectopic gene expression specifically in renal medullary interstitial cells though inducing LoxP based recombination, this mouse line will greatly facilitate our exploration of the physiological roles of renal medullary interstitial cells. Furthermore, as elevated tenascin-C expression is reported to play important roles in pathological conditions such as inflammation, infection and cancer in multiple organs including the kidney [12], [14], [20], this tenascin-C-CreER2 knockin mouse may also provide a valuable tool.
